# Interrelated development of autism spectrum disorder symptoms and eating problems in childhood: a population-based cohort

**DOI:** 10.3389/fped.2023.1062012

**Published:** 2023-05-02

**Authors:** Holly A. Harris, Ivonne P. M. Derks, Peter Prinzie, Anneke Louwerse, Manon H. J. Hillegers, Pauline W. Jansen

**Affiliations:** ^1^ Department of Child & Adolescent Psychiatry/Psychology, Erasmus MC, University Medical Center, Rotterdam, Netherlands; ^2^ Generation R Study, Erasmus MC, University Medical Center, Rotterdam, Netherlands; ^3^ Department of Psychology, Education & Child Studies, Erasmus University Rotterdam, Rotterdam, Netherlands; ^4^ Sophia Children's Hospital, Department of General Pediatrics, Erasmus MC, University Medical Center, Rotterdam, Netherlands

**Keywords:** ASD symptoms, autistic traits, eating problems, food selectivity, picky eating, random-intercept cross-lagged panel model, autism spectrum disorder

## Abstract

Eating problems, such as food selectivity or picky eating, are thought to be an epiphenomenon of autism spectrum disorders (ASD). Yet eating problems are also common in the general pediatric population and overlap with ASD symptoms. However, the temporal association between ASD symptoms and eating problems is poorly understood. This study examines the bidirectional association between ASD symptoms and eating problems across child development, and investigates whether these associations differ by child sex. Participants (*N *= 4,930) were from the population-based Generation R Study. Parents reported their child's ASD symptoms and eating problems using the Child Behavior Checklist at 5 assessments from toddlerhood to adolescence (1.5 to 14 years, 50% girls). A Random Intercept Cross-Lagged Panel Model was used to examine the lagged associations between ASD symptoms and eating problems at the within-person level, controlling for stable, trait-like differences at the between-person level. At the between-person level, there was a strong correlation between ASD symptoms and eating problems (β = .48, 95% CI: 0.38 to 0.57). Controlling for these between-person effects, there was limited evidence for consistent, predictive effects of ASD symptoms and eating problems at the within-person level. Associations did not differ by child sex. Findings suggest that ASD symptoms and eating problems may represent a cluster of traits that are highly stable from early childhood to adolescence, which have a minimal reciprocal effect at the individual-level. Future research could focus on these trait-like qualities to inform the development of supportive, family-focused interventions.

## 1. Introduction

Autism spectrum disorders (ASD) are neurodevelopmental conditions characterized by social communication deficits, and restrictive and repetitive behavioral patterns (1). Some features inherent to ASD, such as impaired sensory processing and rigid behavior, may manifest in eating problems (2). Children with ASD are five times more likely than neurotypical children to have eating problems (3) which may persist into adolescence (2) and possibly young adulthood (4). Indeed, Suarez et al. (5) showed persistence of eating problems over a 20-month time period in children with ASD (*n *= 52). On the other hand, Bandini et al. (6) showed that some aspects of eating problems, such as food refusal or frequency of problematic mealtime behaviors (e.g., tantrums about food), improved over a longer time period from childhood to adolescence (6.8 to 13.2 years, *n *= 18). Eating problems may predispose children with diagnosed and even subclinical ASD symptoms to poor diet quality (7) and suboptimal nutritional intake (8). While eating problems are presumed to be an epiphenomenon of ASD, eating problems may also modulate diet to influence the severity of ASD-associated symptoms (9, 10). Cumulating evidence from population-based cohorts suggest that eating problems observed in infancy and toddlerhood may be an early indicator of elevated ASD symptoms in mid-childhood (11, 12). However, no epidemiological studies to date have investigated the potentially interrelated development of ASD symptoms and eating problems from early childhood to adolescence.

Although eating problems are common in childhood, the definition of eating problems varies widely in the literature (2). Eating problems may be characterized by, although not limited to, food selectivity, food refusal, poor dietary intake or nutrient inadequacy (13), disruptive or problematic mealtime behavior (14), slow eating and food neophobia (15). Food selectivity is the most common eating problem experienced by children with ASD, where only a narrow variety of food is consumed (3). Many neurotypical children also experience eating problems in the preschool years, although this is a temporary phase for most children (16). Yet eating problems are thought to be more prevalent, more severe, and more enduring in children with ASD (3). Regardless of the type, source or duration of eating problems they can be a significant source of concern for parents (17), and could indicate the need for behavioral, nutritional or lifestyle interventions.

What is currently known about the association between ASD and eating problems has generally been derived from studies that compare children with a clinical diagnosis of ASD to neurotypical children. However, there are several limitations to this approach which obfuscate the understanding of ASD and eating problems. Firstly, emerging evidence suggests an overlap between the severity of ASD symptoms and eating problems, like food selectivity, not only at the level of children with clinically-diagnosed ASD (18) but also at the population-level (19). Case-control studies comparing children with diagnosed ASD to neurotypical children tend to overlook the evolving continuum of ASD symptoms in the general population. Secondly, researching children who have already received an ASD diagnosis precludes the testing of temporal associations between ASD symptoms and eating problems as they emerge (20). Recent meta-analytic evidence suggests that some dietary components could play a role in the expression of ASD-associated symptoms in those with ASD (10), although it is unclear whether this finding can be generalized to community-based samples. Finally, while there is a male-bias in ASD prevalence, studying populations with clinically-diagnosed ASD under-represents girls who are relatively underdiagnosed or who may present with different symptoms compared to boys (21). Evidence from population-based samples suggest that ASD symptom trajectories may differ by child sex—typically emerging later in childhood for females—although sex differences may disappear into adolescence (22). Thus, examining children diagnosed with ASD may potentially bias eating-related research to males. A dimensional approach to assessing ASD symptoms in population-based samples over time can shed light on the developmental etiology of ASD and eating problems.

A combination of repeated measures taken throughout child development and appropriate statistical modelling techniques are key in understanding whether eating problems precede ASD symptoms, or vice versa. Testing temporal directionality of associations has traditionally been undertaken using the Cross-Lagged Panel Model (CLPM), which examines the mean-level, lagged associations between constructs within a population (23). However, this model does not disaggregate stable, trait-like between-person differences from state-like, within-person changes over time. The Random Intercept Cross-Lagged Panel Model (RI-CLPM) is an extension of the CLPM that controls for stable, between-person differences to isolate the lagged effect of the temporal deviation from an individual's mean score on one construct to the temporal deviation from their mean score on another construct (23). This statistical approach better aligns with child developmental processes and is growing in popularity in the pediatric literature (24). Exploring the directionality of associations between ASD symptoms and eating problems using the RI-CLPM could be leveraged for hypothesis-generating, building theoretical frameworks and guiding future research to understand when and how (if possible) to best intervene in the course of eating problems and ASD symptoms.

This exploratory, population-based study had two aims. Aim 1 was to investigate whether ASD symptoms predict later eating problems or vice versa within individuals, or whether these behaviors remain stable, yet related, across child development. Aim 2 was to explore whether within-individual lagged associations between ASD symptoms and eating problems differed by child sex. Unravelling these pathways has the potential to inform early identification processes and intervention strategies to support parents in managing eating problems in children with a high-level autistic trait phenotype.

## 2. Methods

### 2.1. Study design and participants

The Generation R Study (25) is a population-based cohort on health and development from fetal life onwards. All pregnant women living in Rotterdam, the Netherlands, with an expected delivery date between April 2002 and January 2006 were invited to participate (*N *= 9,778; participation rate: 61%). Ethical approval was granted by the Medical Ethical Committee of the Erasmus Medical Center Rotterdam. Written informed consent was obtained from parents of all children. Full consent for participation up to the age of 14 years was obtained from *n *= 5,447 children and their parents. The current study uses data collected from 5 waves, when children were 1.5, 3, 6, 10 and 14 years old. Children with ≥2 repeated measures of both eating problems and ASD symptoms were included in this study (*n *= 4,930). Included children were more likely to have a Western ethnicity, greater birth weight, a lower Body Mass Index (BMI) z-score at 14 years; and have older mothers with higher levels of education and a lower pre-pregnancy BMI (all *p *< .001) compared to those excluded.

### 2.2. Measures

An overview of all the measures used at each wave is provided in Supplementary File Table S1.

#### 2.2.1. ASD symptoms

In the current study, we assessed symptoms of ASD rather than clinically diagnosed ASD. At child age 1.5, 3 and 6 years, parents (90%–96% mothers) completed the Child Behavior Checklist (CBCL)/1.5–5 (26). The DSM-Oriented subscale “Pervasive Developmental Problems” (PDP; 13 items) was used as an indicator for ASD symptoms (27). Cohort T-scores of the PDD are available in Supplementary File Table S2. At child age 10 and 14 years, parents completed the CBCL/6–18 (28). However, a standard subscale for assessing ASD symptoms has not been constructed in this version for older children. Two independent studies (29, 30) of Dutch children have shown that elevated scores on 10 items from the CBCL/6–18 can adequately discriminate between children with and without an ASD diagnosis (example item: “*repeats certain acts over and over*”, other items shown in Supplementary File Table S3). To investigate the construct validity of this 10 item ASD symptoms subscale (29) in the current sample, we examined the correlation between this subscale and the 18 item Social Responsiveness Scale (SRS) short form (31). The SRS is an autism screening questionnaire which covers all domains of the DSM-5 ASD diagnostic criteria, including social cognition, social communication and autistic mannerisms (31) and shows good diagnostic validity (32, 33). Parents reported the SRS at child age 14 years. The Pearson's correlation coefficient between the SRS and the 10-item CBCL/6–18 ASD symptoms subscale at 14 years was *r *= .58, *p *< .001. Parents responded to all items on the CBCL on a scale of 0 (*Never*) to 2 (*Often*) and items were averaged to produce mean item ASD symptoms scores at 1.5, 3, 6, 10 and 14 years.

#### 2.2.2. Eating problems

Eating problems in the current study is operationalized as “poor dietary intake” and/or “food refusal”. At child age 1.5, 3 and 6 years, parents reported on their child's eating problems using two items from the CBCL/1.5–5 (26). Parents were asked to indicate how frequently their child “*does not eat well*” or “*refuses to eat*”. Item responses were anchored on a scale of 0 (*Never*) to 2 (*Often*) and items were averaged to produce mean item eating problem scores at 1.5, 3 and 6 years. These items have been used previously in this cohort to characterize picky eating trajectories (16). At child age 10 and 14 years, parents reported on a single item (“*does not eat well*”) from the CBCL/6–18 to indicate eating problems (28). The second eating problem item (i.e., “*refuses to eat*”) was dropped in this version for older children. Therefore, the single item responses were used to assess eating problems from 0 (*Never*) to 2 (*Often*) at these waves, rather than a mean score. Previous research by Prosperi et al. (34) have also used this single CBCL item as a broad indicator of eating problems in children with ASD. For descriptive purposes, we examined correlations between the single eating problem item and an indicator of “food selectivity”, one of the most common eating problems experienced in children with ASD (3). Food selectivity was assessed with the 4-item picky eating subscale from the Stanford Feeding Questionnaire (35). Pearson's correlation coefficients indicated moderate associations between eating problems and food selectivity at 10 (*r *= .37, *p *< .001) and 14 years (*r *= .36, *p *< .001).

#### 2.2.3. Sociodemographic characteristics

Information on child sex, birth weight and gestational age was obtained from hospital/midwife registries. Child ethnicity (Western or non-Western) was based on the country of birth of both biological parents. Children's height and weight were measured by research assistants at the research center visit at 14 years and converted into sex- and age-adjusted BMI z-score using Dutch reference growth curves (36). Mothers' age at enrolment, pre-pregnancy BMI and highest level of education obtained was collected via postal questionnaire during pregnancy.

#### 2.2.4. Statistical analysis

All analyses were carried out in R statistical software, version 4.1.1. Descriptives of the sociodemographic characteristics of the study population are presented in proportions (%) or means. The ASD symptoms and eating problems subscales, even those using 1 item, were treated as continuous scores throughout all the analyses. As a preliminary step, multiple linear regression analyses were used to examine the wave-on-wave longitudinal relationships for both variables separately, e.g., ASD symptoms at 1.5 years predicting eating problems at 3 years, controlling for eating problems at 1.5 years. Covariates were imputed using multiple imputation by chained equations (MICE) using 20 imputed datasets. Pooled linear regressions showed that adjusting for child sex, birthweight, ethnicity, and maternal age at recruitment and education did not significantly alter the results. Therefore, models were not adjusted for covariates in the main analysis to facilitate model parsimony. Correlations and intra-class correlations (ICCs) across ASD symptoms and eating problems were calculated.

To address the first aim, an RI-CLPM analysis was performed to explore the intra-individual, cross-lagged association between ASD symptoms and eating problems from early childhood and adolescence. RI-CLPM analyses disaggregate stable, between-person differences (i.e., trait-like factors) from within-person fluctuations over time by inclusion of a factor with all loadings constrained to 1 (i.e., a random intercept) (23, 37). RI-CLPM analyses were performed using the *lavaan* package (38). Missing data on ASD symptoms and eating problems were handled using Full Information Maximum Likelihood (FIML). To account for the skewed data, parameters were estimated using the Maximum Likelihood estimator with Robust standard errors (MLR). Overall goodness of fit of the models was determined using cut-offs including Tucker-Lewis index (TLI) and the Comparative Fit Index (CFI) values > 0.95; and root-mean-square error of approximation (RMSEA) < 0.06 (39). The time intervals between waves varied throughout the study, therefore, we did not constrain the model to test whether the dynamics of processes are time invariant (37). We additionally adjusted for multiple testing using the Benjamini-Hochberg False Discovery Rate (FDR) to account for the number of parameters tested. In addition to the primary analysis, we ran a multi-group analysis to test if the cross-lagged effects were equal for boys and girls following the procedure outlined by Mulder and Hamaker (37). First, we estimated a multi-group RI-CLPM with unconstrained cross-lagged pathways by child sex using the “group” function in *lavaan*. We then estimated a multi-group RI-CLPM in which the cross-lagged pathways were invariant (e.g., constrained to be equal) across child sex. The model fit of the unconstrained and constrained models was compared using the Satorra–Bentler adjusted *χ*^2^ difference test. We also ran two sensitivity analyses. Firstly, we reran the model using only 1 item assessing eating problem (*“does not eat well”*) so that the measure in waves 1 to 3 were equivalent to the measure in waves 4 and 5. Secondly, we reran the model with eating problems dichotomized at every timepoint as “no eating problems” vs. “any eating problems” (defined by mean score > 0 from ages 1.5 to 6 years or “Sometimes” and “Often” at ages 10 and 14 years), to check whether skewness of the eating problems measure influenced our findings.

## 3. Results

There was an equal proportion of boys to girls in the sample (see Table 1 for sociodemographic characteristics). Most of the children in the sample had a Western background (77%) and had mothers who were university educated (58%). Pearson's correlations showed that ASD symptoms were positively correlated across all 5 waves (rs ranging from .17 to .61), as were eating problems (rs ranging from.08 to.43; see Supplementary File Table S4 for study measures descriptive statistics). Spearman's Rank Order Correlation tests were also run to account for the non-normality of the data, and results were the same as the Pearson correlations. Paired samples Wilcoxon sign-ranked tests were used to examine the wave-on-wave differences in ASD symptoms or eating problems. For ASD symptoms, mean individual scores significantly increased from wave to wave (*p *< 0.05). For eating problems, mean individual scores significantly decreased from wave-to-wave (*p *< 0.01) with the exception of eating problems from 10 to 14 years, which significantly increased (*p *< 0.001).

**Table 1:** Sociodemographic characteristics (*N *= 4,930).

Child	% or M ± SD	*n*
Boys (%)	49.7	2,452
Birth weight, grams (M ± SD)	3,430.7 ± 570.7	4,926
Gestational age, weeks (M ± SD)	39.8 ± 1.8	4,913
Ethnicity (%)		
Western	77.0	3,778
Non-Western	23.0	1,126
BMIz score at 14 years (M ± SD)	0.17 ± 1.1	3,838
**Mother**		
Age at inclusion, years (M ± SD)	31.5 ± 4.6	4,930
Pre-pregnancy BMI (kg/m^2^) (M ± SD)	23.5 ± 3.9	3,729
Highest level of education (%)		
University educated	57.6	2,703
Not university educated	42.4	1,989

Multiple linear regression analyses showed that eating problems were positively associated with ASD symptoms at every subsequent wave while adjusting for baseline ASD symptoms, with the exception of eating problems at 6 years which were not associated with ASD symptoms at 10 years (see Supplementary File Table S5 for the longitudinal multiple linear regression models). ASD symptoms were also associated with eating problems at every subsequent wave while adjusting for baseline eating problems, with the exception of ASD symptoms at 6 years which were not associated with eating problems at 10 years. ICCs showed that 74% of the variance in ASD symptom score was explained by between-person differences (or stable traits). For eating problems, 67% of the variance was explained by between-person differences. Therefore, the RI-CLPM indicated how the within-person fluctuations in ASD symptoms and eating problems (i.e., 26% and 33% of the variance, respectively) predicted each other while controlling for stable, between-person differences.

**Figure 1:**
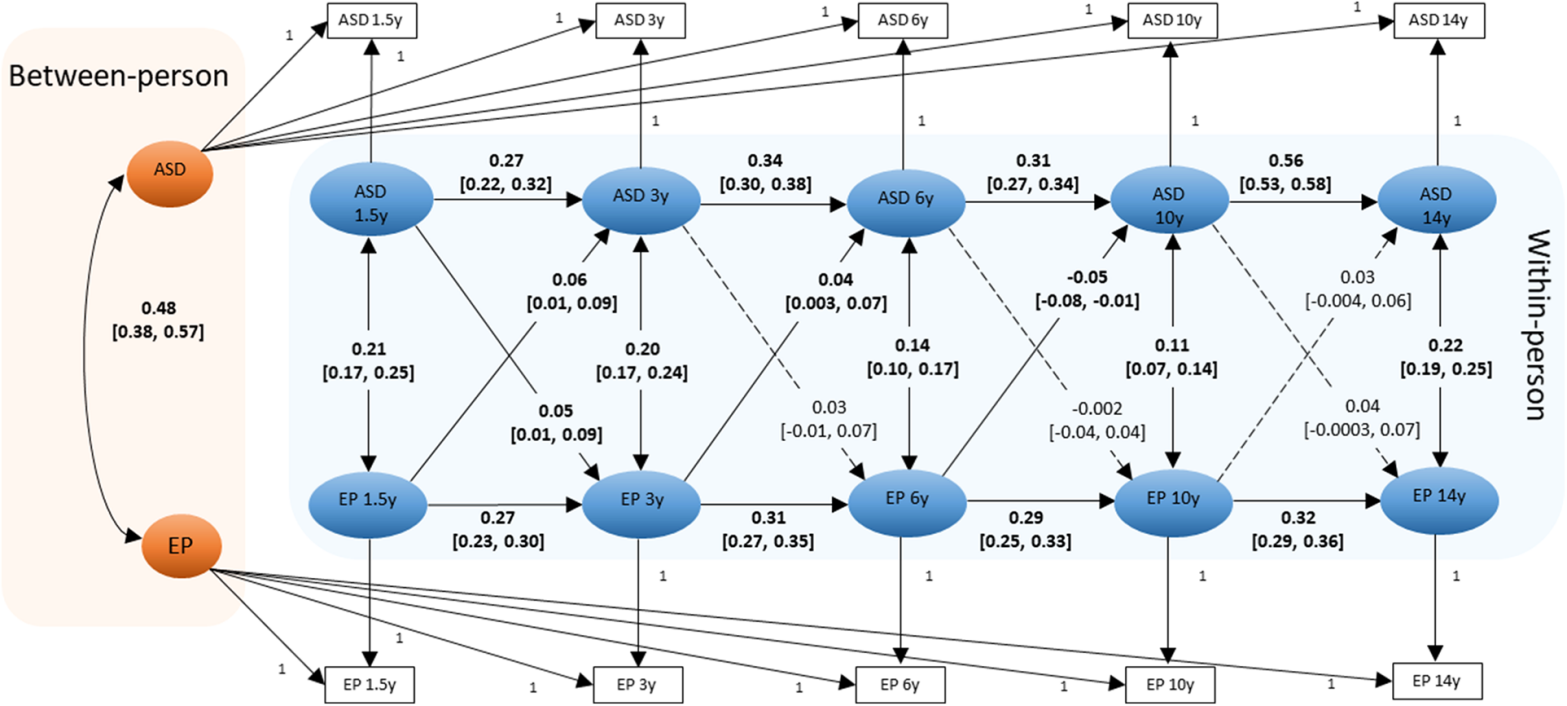
Diagram of the RI-CLPM examining the *between-person* and *within-person effects* between ASD symptoms and eating problems (*N *= 4,930). Standardized beta coefficients and [95% Confidence Intervals] for pathways are shown. Statistically significant paths are shown in solid lines (and coefficients are bold); dotted lines show the non-significant paths. ASD: ASD symptoms; EP: eating problems. Residuals are omitted for clarity of presentation.

The RI-CLPM model fitted well with the data, CFI = 0.984, TLI = 0.966 and RMSEA = 0.036 (Figure 1). At the between-person level, there was a moderate to strong positive association between ASD symptoms and eating problems (β = .48). Therefore, individuals with higher average ASD symptoms scores reported higher average eating problems scores across the 5 measurement waves. At the within-person level, the positive autoregressive effects for ASD symptoms indicate that individuals with an elevated ASD symptom score at one occasion also showed an elevated ASD symptom score at the next occasion, relative to their own average score (e.g., ASD symptoms from 1.5 years to 3 years: β = .27). Similarly, positive autoregressive effects were also observed for eating problems (e.g., eating problems from 1.5 years to 3 years: β = .27). Positive concurrent associations between ASD symptoms and eating problems were also observed, indicating that children who scored higher than their average ASD symptoms score also scored higher than their average eating problems score at the same wave (e.g., concurrent association at 1.5 years: β = .21). Evidence of a small, positive bidirectional association was observed between ASD symptoms and eating problems from 1.5 years to 3 years (e.g., eating problems at 1.5 years to ASD symptoms at 3 years: β = .06). From 3 years to 6 years, there was a positive lagged effect from eating problems to subsequent ASD symptoms (β = .04). From 6 years to 10 years, there was a negative lagged effect from eating problems to ASD symptoms (β = -.05). All associations remained statistically significant after adjusting for multiple testing. Constraining the cross-lagged parameters to be invariant across child sex did not significantly worsen the model fit [*Δχ*^2^(8) = 9.49, *p *= .30]. This indicates that parameter estimates were similar for boys and girls.

In the first sensitivity analysis, associations remained in the same direction when using the 1 item indicator of eating problems in waves 1 to 3. However, some cross-lagged associations changed in significance, likely due to the decreased variation in eating problems and smaller effect sizes. In the second sensitivity analysis whereby eating problems were dichotomized, the results were similar to those of the primary analysis (not shown, but available upon request).

## 4 . Discussion

This is the first study to explore intra-individual, reciprocal associations between ASD symptoms and eating problems in a population-based cohort. Study findings suggest that ASD symptoms and eating problems are highly correlated at the latent-trait level throughout child development. That is, children with higher average ASD symptom scores also had more eating problems from 1.5 to 14 years. Once controlling for the stable, trait-like association between ASD symptoms and eating problems, there was limited evidence for reciprocal longitudinal associations at the intra-individual level between these factors. Findings of lagged associations between ASD symptoms and eating problems should be interpreted in light of their small effect sizes in addition to the large sample size of the study. Toddlers (1.5 years) with higher than their average level of ASD symptoms had higher subsequent eating problems at 3 years, and vice versa. In the preschool years, elevated eating problems at 3 years preceded a subsequent increase in ASD symptoms. In early to mid-childhood, elevated eating problems at 6 years preceded a subsequent decrease in ASD symptoms at 10 years. These within-person, cross-lagged associations did not differ by child sex. It is worth noting that these findings speak to the directionality of parent-reported associations in an observational setting, and firm conclusions about causality cannot be established. Further research is warranted to ascertain whether intervening on eating problems in the general pediatric population, for example, will result in changes in the subsequent expression of ASD symptoms using experimental study designs.

Findings from the current study suggest that ASD symptoms and eating problems are temporally stable and strongly correlated at the population-level. This adds significant knowledge to the current understanding of ASD and eating problems, with implications for research and practice. While it is widely accepted that children with ASD experience disproportionate eating problems (3), our results suggest that subclinical ASD symptoms at the population-level are also associated with more eating problems. ASD symptomology and eating problems may represent a constellation of phenotypes grounded in shared characteristics, such as atypical sensory sensitivities and cognitive rigidity, found in clinical and general populations and across ages (40). Future research could investigate the potential shared etiology of ASD symptoms and eating problems. Such research might include the examination of overlapping heritability of ASD symptoms and eating problems. Furthermore, network analysis could be leveraged to identify the specific phenotypic features common between ASD symptoms (i.e., social and communication problems or repetitive, stereotyped behavior) and eating problems (i.e., food selectivity, or disruptive mealtime behavior). It is important to increase health service providers' awareness of the shared characteristics of ASD features and eating problems and ensure that nutrition-related behaviors are evaluated and communicated within the multidisciplinary team where treatment is accessed. Additionally, it would be useful for providers, when working with families, to frame eating as a complex skill for some children to master, which could require ongoing management through development.

The small bidirectional association between ASD symptoms and eating problems in toddlerhood years suggest the need to further investigate this developmental stage as a window of opportunity for early screening and intervention. The early years mark an important period for brain growth, where atypical connectivity associated with ASD emerges (41). More frequent food refusal is common during this time, although this gradually subsides over time for most children (16). Children with atypical sensory sensitivities, delays in motor development (i.e., oral-motor eating skills), difficulties in communicating their preferences to caregivers and a need for “sameness” may find eating more challenging, which could reinforce early eating problems (11, 12). Eating is also a social activity that is embedded within the daily family routine. Mealtimes could therefore present a tangible platform to identify early ASD-like behaviors and to intervene in problematic eating behaviors. Children with less severe ASD symptoms may be more difficult to identify but may still benefit from nutritional intervention (42). Our results suggest a need to develop and test age-appropriate tools in toddlerhood to assist in identifying specific eating problems which could be indicative of mild ASD, and tailor interventions accordingly. While early interventions typically focus on cognitive, language and behavioral outcomes, eating problems are rarely addressed in early interventions for ASD (43). It is critical to understand if addressing early eating problems improves children's and parents' ability to cope with atypical sensory sensitivity and the social demands of mealtimes.

The unidirectional findings of eating problems on subsequent ASD symptoms from 3 years to 10 years were surprising and are difficult to explain. More eating problems at 3 years predicted a subsequent increase in ASD symptoms at 6 years, which may reflect that eating problems could be an indicator of developmental delay during this time period. Alternatively, this finding could be suggestive of a relation in which poor eating habits may exacerbate ASD symptoms. This speculation is in line with research showing the role of diet in influencing the severity of ASD-associated symptoms (9, 10). However, more eating problems at 6 years predicted a subsequent *decrease* in ASD symptoms at 10 years. The most likely explanation for this peculiar finding may be related to the change in CBCL measures between these two waves. This change in CBCL measure may also explain the lack of a significant longitudinal association of ASD symptoms at 6 years with later eating problems. Changes in measurement tools used is an undeniable, major limitation of the current study. That said, a change in the direction of an effect is possible when disaggregating between-person from within-person processes (24). If this is the case, a potential explanation for this finding could be related to how families manage or accommodate eating problems, or could reflect families prioritizing treatment-seeking in other areas related to core autistic traits (such as academia). Nevertheless, we strongly recommend replication of this finding using identical, repeated measures across waves to support or refute this association.

Limitations of this study include the use of parent-reported measures, and some inconsistency in measures of ASD symptoms and eating problems. Although a moderate correlation between the recently developed ASD symptoms scale and the SRS was observed (31), the internal reliability for the ASD symptom scale was below acceptable levels (29). Furthermore, the test-retest reliability of this scale has not been examined and further research is required to examine longitudinal measurement invariance over time. Moreover, we used a broad indicator of eating problems, which was only moderately correlated with the “picky eating” subscale from the Stanford Feeding Questionnaire. We suggest exercising caution when interpreting the results, and future research could consider specifying the types of eating problems, which may be age- and sex-dependent (12, 44). Finally, findings may not be applicable to clinical ASD cohorts with potentially different trajectories of symptom severity and eating problems (45). Strengths of our study include the novel exploration of reciprocal associations between ASD symptoms and eating problems at the between- and within-person level. The large sample size enabled us to detect small effect sizes. However, the clinical significance of these small effect sizes in the general population is unknown, and it is possible that small mean differences, particularly early in life, may reflect larger individual differences (46). Finally, we also took a dimensional approach to investigating ASD symptoms with a continuous indicator, therefore providing more information about the broader autism spectrum.

This is the first exploratory study to begin unravelling pathways of development between ASD symptoms and eating problems in the general pediatric population. ASD symptoms and eating problems appear to be mainly stable and highly correlated traits from ages 1.5 to 14 years. This suggests that ASD symptoms and eating problems may form part of a cluster of phenotypes based on underlying, shared characteristics. There was limited evidence for an intra-individual associations between ASD symptoms and eating problems. Replication of these findings are recommended, and further research is required to understand to the potentially shared etiology of ASD symptoms and eating problems in community-based samples.

## Data Availability

The data analyzed in this study is subject to restrictions. Data described in the manuscript, code book, and analytic code can be made available upon request to datamanagementgenr@erasmusmc.nl and will be discussed in the Generation R Study Management Team.
