# Supplementary material for: Interrelated development of autism spectrum disorder symptoms and eating problems in childhood: a population-based cohort
Source: Front Pediatr. 2023 May 2;11:1062012. doi: 10.3389/fped.2023.1062012 (PMC10185905; doi:10.3389/fped.2023.1062012)
Supplement: Supplementary file 1 [file Datasheet1.pdf]

Table S1. Measures used at each wave for ASD symptoms and eating problems

Wave (child age)	ASD Symptoms	Eating problems
	(subscale, questionnaire, number of items)	
1 (1.5 years)	Pervasive Developmental Problems CBCL/1.5-5, 13 items	Eating problems ("does not eat well" and "refuses to eat"), CBCL/1.5-5, 2 items
2 (3 years)	Pervasive Developmental Problems CBCL/1.5-5, 13 items	Eating problems ("does not eat well" and "refuses to eat"), CBCL/1.5-5, 2 items
3 (6 years)	Pervasive Developmental Problems CBCL/1.5-5, 13 items	Eating problems ("does not eat well" and "refuses to eat"), CBCL/1.5-5, 2 items
4 (10 years)	ASD symptoms subscale by So et al. (2013), CBCL/6-18, 10 items	Eating problems ("does not eat well"), CBCL/6-18, 1 item
5 (14 years)	ASD symptoms subscale by So et al. (2013), CBCL/6-18, 10 items	Eating problems ("does not eat well"), CBCL/6-18, 1 item

CBCL: Child Behavior Checklist

Table S2. Sample T-scores for the Pervasive Developmental Problems scale of the CBCL/1.5-5

				Percentile		
Wave	Age (y)	*n*	M ± SD	25th	50th	75th
T1[a]	1.5	4211	52 ± 4	50	50	51
T2[a]	3	4177	52 ± 5	50	50	51
T3[a]	6	4501	52 ± 5	50	50	51

CBCL: Child Behavior Checklist; y: years.

Table S3. Item numbers and items for the ASD symptoms subscale in the CBCL/6-18, validated by So et al. (2013)

#	Item	Corresponding CBCL scale
1	Acts too young for his/her age	Attention Problems
9	Cannot get his/her mind off certain thoughts; obsessions	Thought Problems
17	Daydreams or gets lost in his/her thoughts	Attention Problems
42	Would rather be alone than with others	Withdrawn/Depressed
62	Poorly coordinated or clumsy	Social Problems
66	Repeats certain acts over and over	Thought Problems
79	Speech problem	Social Problems
80	Stares blankly	Attention Problems
84	Strange behavior	Thought Problems
111	Withdrawn, does not get involved with others	Withdrawn/Depressed

CBCL: Child Behavior Checklist

Table S4. Descriptives of child ASD symptoms and eating problem constructs

Wave	Age (y)	*n*	M ± SD	Percentile 25th	50th	75th	α	Pearson correlation, *r*			
**ASD symptoms**								T2[a]	T3[a]	T4[b]	T5[b]
T1[a]	1.5	4211	0.13 ± 0.15	0	0.08	0.17	.64	.43	.31	.17	.19
T2[a]	3	4177	0.15 ± 0.17	0	0.08	0.23	.68	-	.48	.26	.26
T3[a]	6	4501	0.16 ± 0.18	0	0.15	0.23	.71	-	-	.41	.38
T4[b]	10	4009	0.16 ± 0.19	0	0.10	0.2	.66	-	-	-	.61
T5[b]	14	3927	0.18 ± 0.20	0	0.10	0.3	.68	-	-	-	-
**Eating problems**								T2[c]	T3[c]	T4[d]	T5[d]
T1[c]	1.5	4219	0.40 ± 0.49	0	0	1	.75	.37	.22	.13	.08
T2[c]	3	4155	0.42 ± 0.51	0	0	1	.76	-	.42	.27	.19
T3[c]	6	4497	0.26 ± 0.43	0	0	0.5	.68	-	-	.42	.31
T4[d]	10	4021	0.20 ± 0.45	0	0	0	-	-	-	-	.43
T5[d]	14	3940	0.24 ± 0.49	0	0	0	-	-	-	-	-

Potential range for all constructs is 0 to 2. All Pearson correlations (*r*) significant at the $p<.001$ level. Spearman's Rank Order Correlation tests were run to account for the non-normality of the data, and results were the same. Children were included for analysis if they had a least 2 repeated measures of ASD symptoms and eating problems; *n* participants with measures available for each time point; α=Cronbach alpha (calculated for scales with >1 item); All measures are derived from the Child Behavior Checklist (CBCL/1.5-5, CBCL/6-18); Subscales are: [a]Pervasive Developmental Problems; [b]ASD symptoms ; [c]Eating problems (2 items); [d]Eating problems (single item: *"Does not eat well"*)

Table S5. Multiple linear regressions between ASD symptoms and eating problems

Outcome	Determinant	β	(95% CI)	R^2
ASD symptoms - 3y[a]	Eating problems – 1y[c]	0.06	(0.03, 0.09)	0.19
	ASD symptoms – 1y[a]	0.43	(0.19, 0.40)	
ASD symptoms - 6y[a]	Eating problems – 3y[c]	0.05	(0.02, 0.08)	0.24
	ASD symptoms – 3y[a]	0.48	(0.24, 0.45)	
ASD symptoms - 10y[b]	Eating problems – 6y[c]	-0.01	(-0.04, 0.02)	0.17
	ASD symptoms – 6y[a]	0.42	(0.39, 0.45)	
ASD symptoms - 14y[b]	Eating problems - 10y[d]	0.05	(0.02, 0.07)	0.38
	ASD symptoms – 10y[b]	0.60	(0.58, 0.63)	
Eating problems - 3y[c]	ASD symptoms – 1y[a]	0.09	(0.06, 0.12)	0.15
	Eating problems – 1y[c]	0.36	(0.33, 0.39)	
Eating problems - 6y[c]	ASD symptoms – 3y[a]	0.05	(0.01, 0.09)	0.18
	Eating problems – 3y[c]	0.40	(0.36, 0.44)	
Eating problems - 10y[d]	ASD symptoms – 6y[a]	0.03	(-0.005, 0.06)	0.18
	Eating problems – 6y[c]	0.41	(0.38, 0.44)	
Eating problems - 14y[d]	ASD symptoms – 10y[b]	0.06	(0.03, 0.09)	0.18
	Eating problems – 10y[d]	0.42	(0.39, 0.45)	

Values represent unadjusted standardized coefficients (β), 95% Confidence Intervals (CI) and R^2 for each model; N for each analysis is dependent on available data on the main variables of interest within 2 waves. All measures are derived from the Child Behavior Checklist (CBCL/1.5-5, CBCL/6-18); Subscales are: [a]Pervasive Developmental Problems; [b]ASD symptoms; [c]Eating problems (2 items); [d]Eating problems (single item: *"Does not eat well"*)
